# Luteolin alleviates oxygen-glucose deprivation/reoxygenation-induced neuron injury by regulating NLRP3/IL-1β signaling

**DOI:** 10.1515/med-2025-1198

**Published:** 2025-10-27

**Authors:** Fei Yu, Guangxue Wang, Xingyi Chen, Yanfei Zhang, Cheng Yang, Hui Hu, Liang Wei

**Affiliations:** Department of Neurology, East Hospital, Tongji University School of Medicine, Shanghai, 200120, China; Research Center for Translational Medicine, East Hospital, Tongji University School of Medicine, Shanghai, 200120, China; Department of Medical Department, East Hospital, Tongji University School of Medicine, Shanghai, 200120, China; Department of Neurosurgery, East Hospital, Tongji University School of Medicine, Shanghai, 200120, China

**Keywords:** ischemia/reperfusion, luteolin, NLRP3/IL-1β signaling

## Abstract

We aimed to investigate the protective effect of luteolin against neuron injury induced by oxygen-glucose deprivation/reoxygenation (OGD/R), and to further elucidate the roles of NLRP3 in luteolin-mediated regulation of neuron injury. Using Schwann (SW) 10 cells, an OGD/R-induced neuron injury model was established, and six experimental groups were designated. Subsequently, cell viability and apoptosis were respectively detected by cell counting kit 8 and flow cytometry. Reactive oxygen species (ROS) levels were measured via flow cytometry with a ROS assay kit. Moreover, the expression of interleukin (IL)-6, IL-1β, NLRP3, and MMP9 was examined by real-time quantitative PCR and Western blot. Compared with control cells, OGD/R significantly reduced cell viability and increased apoptosis, ROS levels, and the mRNA levels of *IL-6*, *IL-1β*, *NLRP3*, and *MMP9*. Luteolin significantly enhanced OGD/R-induced cell viability and alleviated apoptosis in SW10 cells (*P* < 0.05). Additionally, luteolin suppressed ROS levels, along with the expression of IL-1β, IL-6, NLRP3, and MMP9 induced by OGD/R. Furthermore, BMS-986299 significantly decreased the cell viability and increased the expression of inflammatory factors in OGD/R-induced SW10 cells treated with luteolin. This inhibitory effect was reversed by NLRP3 knockdown. In conclusion, luteolin may exert a protective effect on OGD/R-induced nerve injury by inhibiting the NLRP3/IL-1β signaling pathway.

## 1 Introduction

Cerebral ischemia is associated with various severe conditions, such as stroke, cardiac arrest, and respiratory arrest [1]. Therapeutic strategies typically center on the rapid restoration of blood flow. However, the restoration of blood circulation can trigger oxidative stress and inflammation damage in regions affected by hypoxia and nutrient deprivation. Moreover, cerebral ischemia–reperfusion (I/R) can lead to impairments in mitochondrial oxidative metabolism and energy depletion within neurons, ultimately resulting in cell death [2,3]. Consequently, the restoration of blood flow following cerebral ischemia may cause additional harm, referred to as I/R injury [4,5]. Therefore, suitable drugs are necessary to safeguard neurons from the impact of I/R injury and mitigate the associated pathological responses [6]. Nevertheless, to date, only a limited number of drugs are available for the clinical treatment of cerebral ischemia [6].

Luteolin, a dietary flavone abundant in numerous plants has been demonstrated to penetrate the brain and exert significant neuroprotective effects [7]. In the context of cerebral I/R, neuroinflammation plays a pivotal role in immune defense through the activation of microglia, an increase in pro-inflammatory mediators, and the promotion of inflammatory cell proliferation [8]. Luteolin has been reported to reduce infarct size and neutrophil accumulation in the ischemic myocardium [9,10,11]. Additionally, it exhibits robust antioxidant and anti-neuroinflammatory effects by inhibiting reactive oxygen species (ROS) and inflammatory factors in cerebral I/R injury [12]. Recently, luteolin has been shown to exert its neuroprotective effects via modulation of various signaling pathways [13,14,15]. However, the precise neuroprotective mechanisms of luteolin against oxygen–glucose deprivation/reoxygenation (OGD/R)-induced neuronal damage remain to be elucidated.

The NOD-like receptor pyrin domain-containing protein 3 (NLRP3) inflammasome is a protein complex. Its activation results in the secretion of the pro-inflammatory cytokine interleukin (IL)-1β [16]. This pathway has been demonstrated to play a substantial role in neuroinflammation and neuronal damage [17]. Activation of the NLRP3 inflammasome can be induced by various cellular stressors, including mitochondrial dysfunction, and oxidative stress, both of which are hallmarks of OGD/R-induced neuronal injury [18,19,20]. Interestingly, recent research has indicated that luteolin may suppress the activation of the NLRP3 inflammasome. However, whether luteolin can exert its neuroprotective effects by modulating the NLRP3/IL-1β signaling pathway in the setting of OGD/R-induced neuronal injury remains uncertain.

Consequently, this study aimed to explore the protective effect of luteolin on OGD/R-induced neuronal injury and to further illuminate the underlying mechanisms, with a specific emphasis on the NLRP3/IL-1β signaling pathway. We hypothesized that luteolin could attenuate neuronal injury by inhibiting the activation of the NLRP3 inflammasome and reducing the production of IL-1β. The findings of this study may contribute to a deeper understanding of the neuroprotective mechanisms of luteolin and identify its potential as a protective agent against brain I/R injury.

## 2 Materials and methods

### 2.1 Cell culture

Schwann (SW) 10 cells were procured from FuHeng Biology (Shanghai, China). SW10 cells, a type of SW cell originating from mouse neural tissue, are also referred to as neuronal SW cells. These cells belong to the glial cell type in the peripheral nervous system, where their primary functions include providing support and protection to neurons, as well as participating in nerve regeneration subsequent to nerve injury. The cells were cultured in Dulbecco’s Modified Eagle’s Medium containing 10% fetal bovine serum (Gibco, USA) and 1% penicillin–streptomycin antibiotics (Gibco) in an incubator maintained at 37°C with 5% CO_2_.

### 2.2 Cell transfection

Small interference (si)-RNA-targeting NLRP3 (si-NLRP3) and si-negative control (si-NC) were obtained from Yanzai Biotechnology (Shanghai, China). The sequences of si-NLRP3-1/2/3 and si-NC are presented as follows: si-NLRP3-1, sense 5′-CCGGCCUUACUUCAAUCUGUUTT-3′, antisense 5′-AACAGAUUGAAGUAAGGCCGGTT-3′; si-NLRP3-2, sense 5′-CCAGGAGAGAACCUCUUAUUUTT-3′, anti-sense 5′-AAAUAAGAGGUUCUCUCCUGGTT-3′; si-NLRP3-3, sense 5′-CCCGGACUGUAAACUACAGAUTT-3′, antisense 5′-AUCUGUAGUUUACAGUCCGGGTT-3′; and si-NC, sense 5′-UUCUCCGAAGGUGUCACGUTT-3′, antisense 5′-ACGUGACACGUUCGGAGAATT-3′. Briefly, SW10 cells at a density of 2 × 10^4^ cells/well were seeded into 24-well plates. Subsequently, 15 pmol of either si-NLRP3 or si-NC was transfected into the cells using Lipofectamine 2000 (Thermo, USA). After 6 h, the medium was replaced with a complete medium. Following an additional 12 h incubation, the transfection efficiency was evaluated by determining the expression of NLRP3 using real-time quantitative PCR (RT-qPCR) and Western blot analysis.

### 2.3 OGD/R induction

SW10 cells were plated and cultured for 24 h, and the original medium was removed on the second day. After being cleaned twice with sugar-free Earle’s solution (EBSS solution; Servicebio, Wuhan, China), EBSS solution was added for maintenance. The cell culture dish was exposed to CoCl_2_ (0, 50, 100, 200, 400, 600 μM; Aladdin, Shanghai, China) to induce chemical hypoxia and placed in a constant temperature incubator (oxygen glucose deprivation, simulating ischemia and hypoxia *in vitro*). After 2 h, the EBSS solution was removed, and cells were maintained in the original culture medium for normal growth (oxygen glucose recovery, simulating reperfusion *in vitro*). The OGD/R-induced SW10 cells were constructed for follow-up experiments.

### 2.4 Grouping

To determine the optimal concentrations of CoCl_2_, luteolin, and NLRP3/IL-1β pathway agonist (BMS-986299), different concentrations of CoCl_2_ (0, 50, 100, 200, 400, and 600 μM), luteolin (0, 1, 2, 5, 10, 20, 50, and 100 μM, Yuanye Bio-Technology Co., Ltd., Shanghai, China), and BMS-986299 (0, 0.2, 0.5, 1, 2, and 5 μM, MedChemExpress, USA) were, respectively, used to treat SW10 cells for 48 h, and then cell viability was detected. Further to explore the effects of luteolin on the growth of SW10 cells induced by OGD/R, the cells were grouped as follows: control, OGD/R, and OGD/R + luteolin groups.

To explore the roles of NLRP3 in luteolin-mediated regulation of cell growth following OGD/R, and the associated mechanisms, the cells were divided into five groups: OGD/R, OGD/R + si-*NLRP3*, OGD/R + luteolin, OGD/R + luteolin + BMS-986299, and OGD/R + luteolin + BMS-986299 + si-*NLRP3* groups.

### 2.5 Cell counting kit-8 (CCK-8) assay

Cells subjected to different treatments were harvested, and 10 μL of CCK-8 solution (Beyotime, Shanghai, China) was added. After 2 h of incubation (with the optical density [OD] maintained at ≤2.0), the absorbance at 450 nm was measured using a Multiskan MK3 (Thermo, USA), and the cell viability curves were drawn.

### 2.6 Apoptosis assays

Cells from each group were digested with trypsin. After adding medium, the cells were gently pipetted to dislodge them and then transferred into the centrifuge tube. After centrifugation at 1,000 rpm for 5 min, the supernatant was discarded. Next, 195 μL of Annexin V-FITC binding solution was added, followed by 5 μL of Annexin V-FITC and propidium iodide staining. Then, the cells were incubated at 5°C for 20 min in the dark. Fluorescence-activated apoptotic cells were analyzed using a flow cytometer (FACSCalibur, BD Biosciences, USA).

## 3 Determination of ROS contents

The levels of ROS in the different groups were measured using a flow cytometry in conjunction with a ROS assay kit (chemical fluorescence method, Nanjing Jiancheng Bioengineering Institute, Nanjing, China) according to the manufacturer’s instructions. Briefly, the cells were centrifuged at 1,000 rpm for 5 min and were resuspended in PBS containing 10 μM DCFH-DA probes. The cells were then cultured at 37°C for 60 min. The DCFH-DA-labeled cells were centrifuged again at 1,000 rpm for 5 min, and the supernatant was removed. After washing twice with PBS, the cell pellets were collected and resuspended in PBS for flow cytometry analysis.

### 3.1 RT-qPCR

Total RNA was extracted from SW10 cells with different treatments using RNAiso Plus (Takara, Dalian, China). Following reverse transcription, the mRNA expressions of *IL-1β*, *IL-6*, *NLRP3*, and matrix metallopeptidase 9 (*MMP9*) were detected using 2× Universal SYBR Green Fast qPCR Mix (ABclonal, USA), with glyceraldehyde-3-phosphate dehydrogenase (GAPDH) as an internal reference. The relative expression levels were calculated using the 2^−∆∆CT^ method. All primer sequences are presented in Table 1.

**Table 1:** The primers used in this study

Gene	Primer sequence (5′→3′)
IL-6-mF	TAGTCCTTCCTACCCCAATTTCC
IL-6-mR	TTGGTCCTTAGCCACTCCTTC
IL-1β-mF	TGCCACCTTTTGACAGTGATG
IL-1β-mF	TGATGTGCTGCTGCGAGATT
NLRP3-mF	ATTACCCGCCCGAGAAAGG
NLRP3-mR	TCGCAGCAAAGATCCACACAG
MMP9-mF	CTGGACAGCCAGACACTAAAG
MMP9-mF	CTCGCGGCAAGTCTTCAGAG
GAPDH-mF	GGTGAAGGTCGGTGTGAACG
GAPDH-mR	CTCGCTCCTGGAAGATGGTG

### 3.2 Western blot

For protein extraction, 200 μL of RIPA lysis buffer (Beyotime) was added to SW10 cells. The protein content was determined using the bicinchoninic acid (BCA) method (Servicebio, USA). Subsequently, the proteins were separated by 10% sodium dodecyl sulfate-polyacrylamide gel electrophoresis and then transferred onto polyvinylidene difluoride membranes. After blocking with 5% non-fat milk powder, the membranes were incubated overnight at 4°C with primary antibodies, including MMP9 (1:1,000; 10375-2-AP; Proteintech, USA); NLRP3 (1:2,000; 68102-1-Ig; Proteintech); IL-6 (1:1,000; 26404-1-AP; Proteintech); IL-1β (1:800; A16288; ABclonal); GAPDH (1:50,000; 60004-1-Ig; Proteintech). The next day, the membranes were incubated with the appropriate secondary antibodies (goat anti-rabbit IgG [H + L]-HRP or goat anti-mouse IgG [H + L]-HRP; 1: 10,000, 111-035-003 or 115-035-003; Jackson ImmunoResearch, USA) for 2 h at 25°C. Finally, the membranes were developed using an enhanced chemiluminescence detection kit (Beyotime).

### 3.3 Statistical analysis

Each experiment was repeated three times, and data were expressed mean ± standard deviation. Statistical analyses were analyzed by GraphPad Prism 5 (GraphPad Software, USA), and the comparison between groups was analyzed by one-way analysis of variance, with *P* < 0.05 as the threshold.

## 4 Results

### 4.1 Selection of optimal concentrations of CoCl_2_, luteolin, and BMS-986299

**Figure 1:**
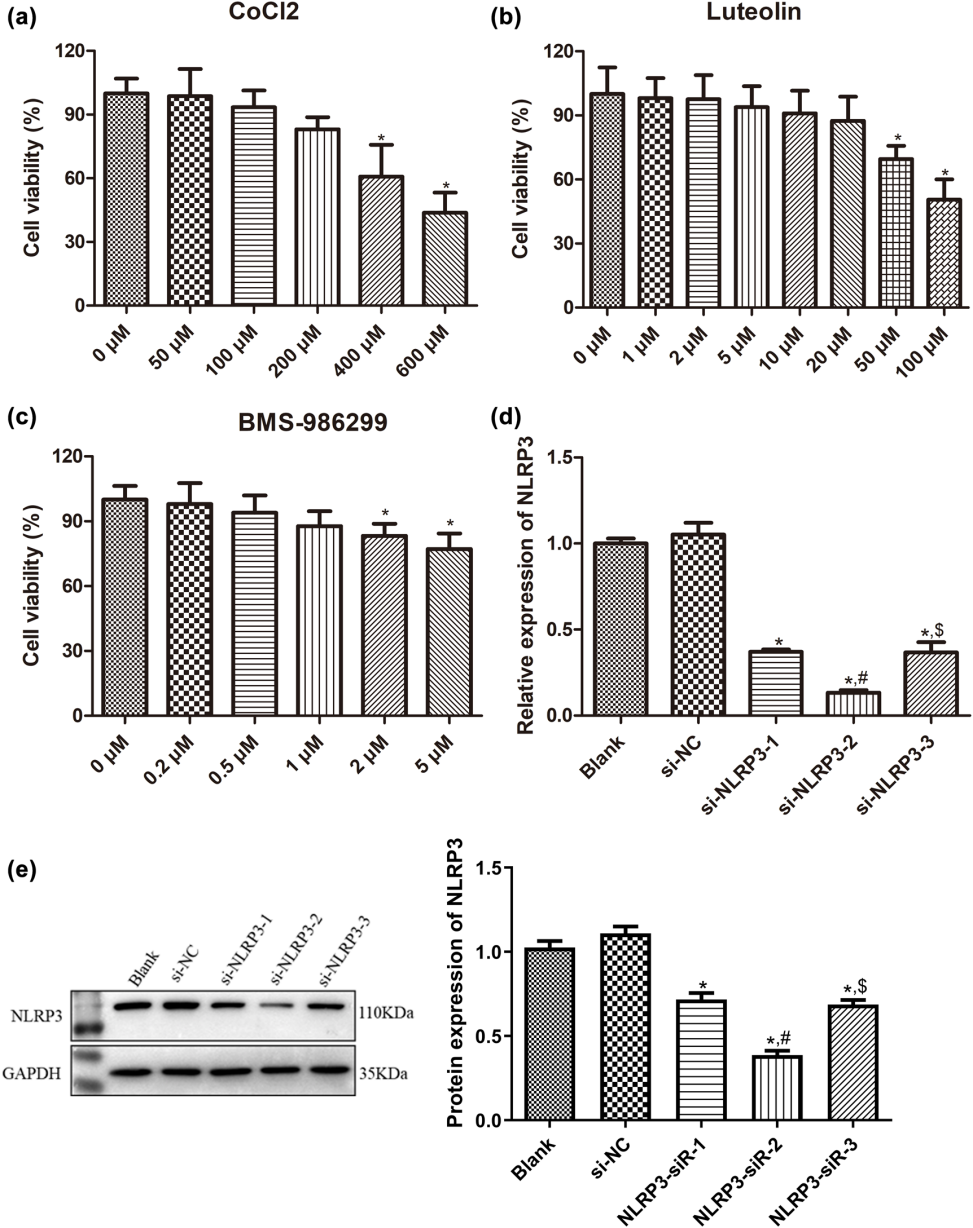
Selection of optimal concentrations of CoCl_2_, luteolin, and BMS-986299. (a) Selection of optimal concentrations of CoCl_2_ by CCK-8. *N* = 4. (b) Selection of optimal concentrations of luteolin by CCK-8. *N* = 4. (c) Selection of optimal concentrations of BMS-986299 by CCK-8. *N* = 4. NLRP3 expression determined to evaluate the cell transfection efficiency using RT-qPCR (d) and Western blot (e). *N* = 3. *P < 0.05 compared with 0 μM group or blank group. ^#^ *P* < 0.05 compared with si-NLRP3-1; ^$^ *P* < 0.05 compared with si-NLRP3-2.

The rational behind choosing the optimal concentrations of CoCl_2_, luteolin, and BMS-986299 is to select a drug concentration that, while not significantly affecting cell viability, can maximize its biological effects. For the selection of the optimal concentration of CoCl_2_, the concentrations of 50, 100, and 200 μM had no apparent impact on cell viability (*P* > 0.05), whereas concentrations of 400 and 600 μM significantly suppressed cell viability (*P* < 0.05, Figure 1a). Consequently, 200 μM of CoCl_2_ was selected for subsequent experiments.

Likewise, luteolin at concentrations of 1, 2, 5, 10, and 20 μM did not significantly influence the viability of SW10 cells (*P* > 0.05), but concentrations of 50 and 100 μM significantly inhibited cell viability (*P* < 0.05, Figure 1b). Therefore, 20 μM of luteolin was selected for further experiments.

When SW10 cells were treated with 0, 0.2, 0.5, and 1 μM of BMS-986299, the cell viability was not significantly affected (*P* > 0.05). However, 2 and 5 μM of BMS-986299 significantly inhibited the cell viability (*P* < 0.05, Figure 1c). Ultimately, 1 μM of BMS-986299 was chosen for subsequent experiments.

### 4.2 Evaluation of cell transfection efficiency

Following the transfection of cells with si-NLRP3, the cell transfection efficiency was assessed using RT-qPCR and Western blot. As depicted in Figure 1d and e, no significant difference was observed in the NLRP3 expression between the blank and si-NC groups (*P* > 0.05); as well as the relative expression of NLRP3 decreased significantly after transfection with si-NLRP3 (*P* < 0.05) compared to the blank group. Specifically, si-NLRP3-2 exhibited the highest transfection efficiency. Consequently, si-NLRP3-2 was chosen for the subsequent experiments.

### 4.3 Effects of luteolin on the growth of SW10 cells induced by OGD/R and its related mechanisms

**Figure 2:**
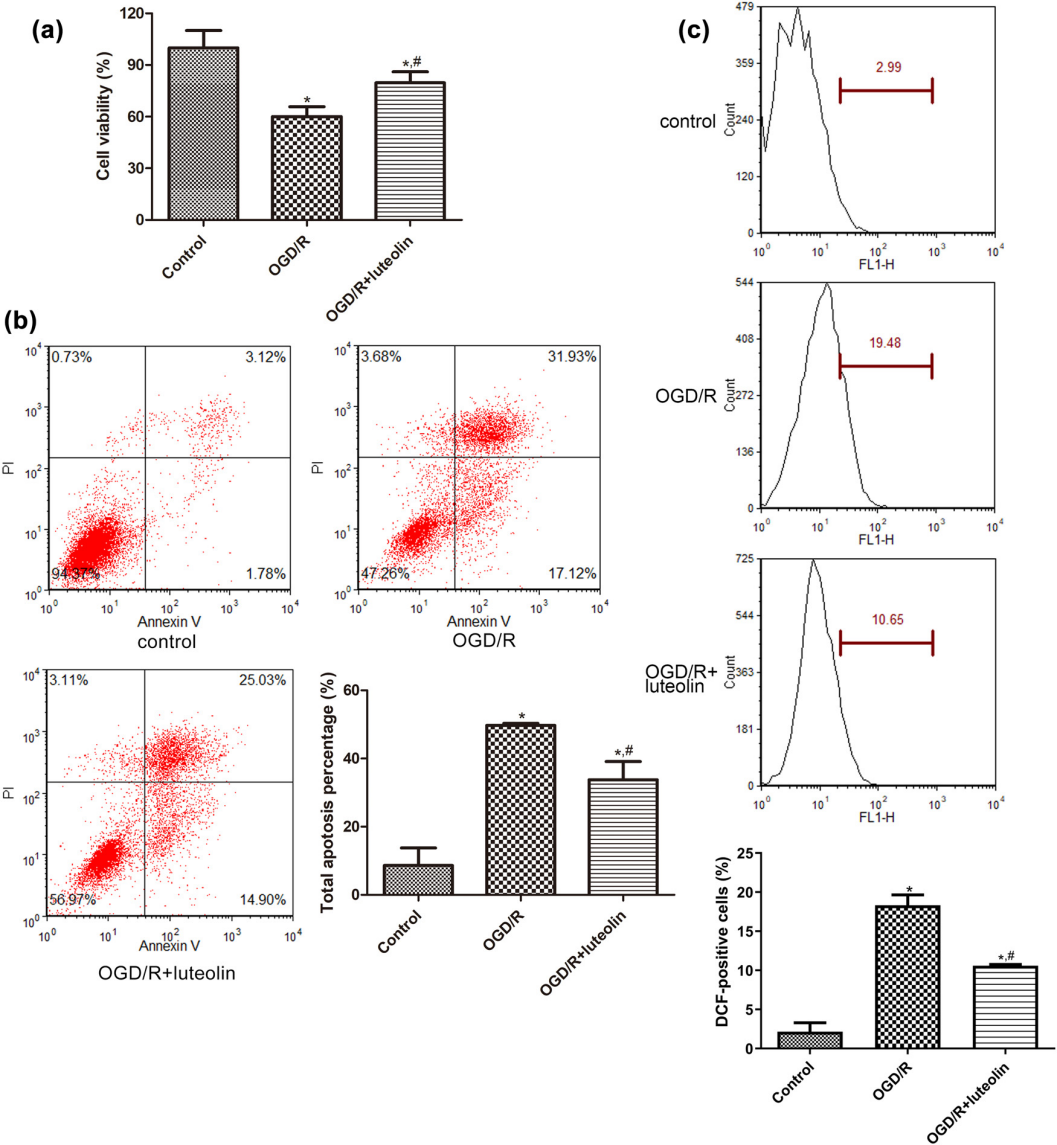
Effects of luteolin on the growth of SW10 cells and on the ROS contents. (a) Cell viability of SW10 cells after OGD/R or treated with luteolin detected by CCK-8. *N* = 4. (b) Apoptosis rate of SW10 cells after OGD/R or treated with luteolin detected by flow cytometry. *N* = 3. (c) The ROS levels in the SW10 cells after OGD/R or treated with luteolin detected by flow cytometry. *N* = 3. *P < 0.05 compared with control. ^#^ *P* < 0.05 compared with OGD/R group.

To investigate the effects of luteolin on the growth of SW10 cells induced by OGD/R, the viability and apoptosis of SW10 cells were measured. It was found that, in comparison to the control group, OGD/R significantly suppressed cell viability (*P* < 0.01), while after the SW10 cells were treated with luteolin, the cell viability was elevated significantly compared to the OGD/R group (*P* < 0.05, Figure 2a). Additionally, when compared to the control cells, the apoptosis rate of SW10 cells increased significantly following OGD/R induction (*P* < 0.01). In contrast, treatment with luteolin significantly decreased the apoptosis rate of SW10 cells relative to the OGD/R group (*P* < 0.05, Figure 2b).

**Figure 3:**
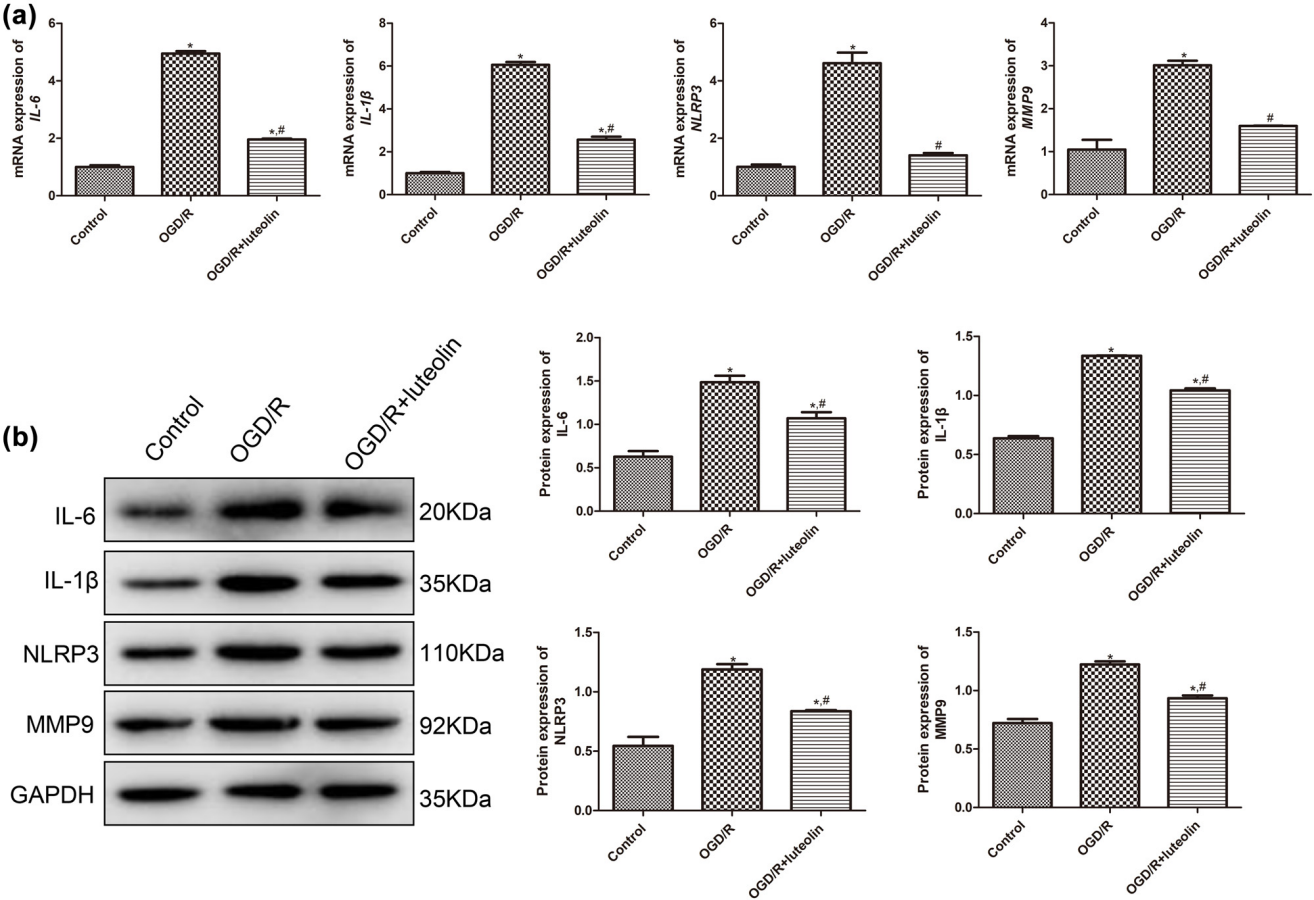
Effects of luteolin on the expression of inflammatory factors in SW10 cells. The expression of IL-6, IL-1β, NLRP3, and MMP9 after OGD/R or treated with luteolin detected by RT-qPCR (a) and Western blot (b). *N* = 3. *P < 0.05 compared with control. ^#^ *P* < 0.05 compared with OGD/R group.

Further, we explored the potential mechanisms by which luteolin regulates OGD/R-induced SW10 cells. Compared with the control group, the ROS level in the OGD/R-induced cells was significantly elevated (*P* < 0.05); whereas luteolin evidently reduced the ROS levels caused by OGD/R (*P* < 0.05, Figure 2c). In addition, OGD/R treatment significantly increased the mRNA expression of *IL-6*, *IL-1β*, *NLRP3*, and *MMP9* in SW10 cells (*P* < 0.05, Figure 3a). Conversely, luteolin evidently reduced the mRNA levels of *IL-6*, *IL-1β*, *NLRP3*, and *MMP9* compared to the OGD/R group (*P* < 0.05). Western blot analysis further confirmed the results of RT-qPCR (Figure 3b).

### 4.4 The roles of NLRP3 in luteolin-mediated regulation of cell growth induced by OGD/R and its related mechanisms

**Figure 4:**
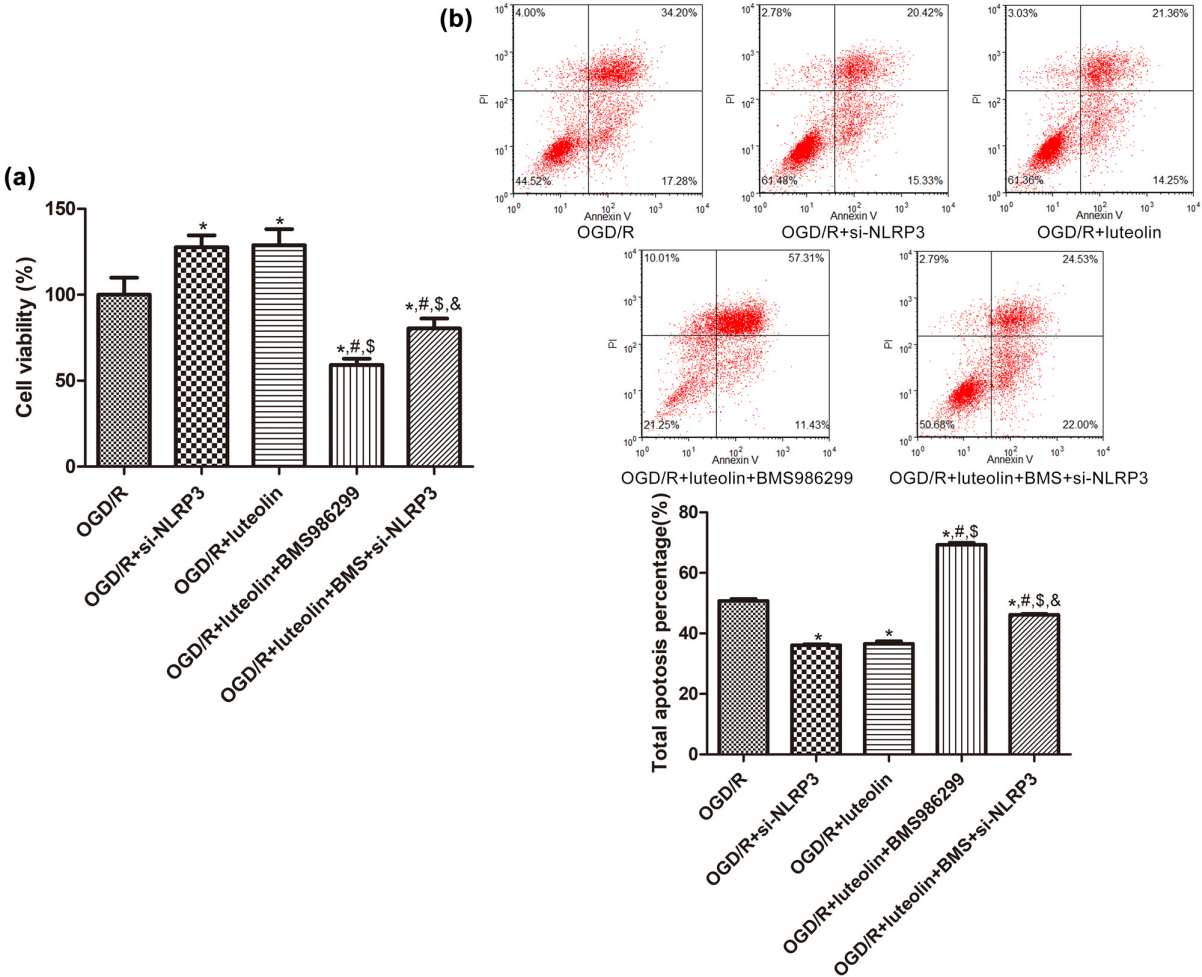
The roles of NLRP3 in luteolin regulating cell growth induced by OGD/R. (a) Cell viability of SW10 cells after OGD/R, NLRP3 knocking down or treated with BMS-986299 detected by CCK-8. *N* = 4. (b) Apoptosis rate of SW10 cells after OGD/R, NLRP3 knocking down or treated with BMS-986299 detected by flow cytometry. *N* = 3. *P < 0.05 compared with OGD/R group. ^#^ *P* < 0.05 compared with OGD/R + si-NLRP3 group. ^$^ *P* < 0.05 compared with OGD/R + luteolin group. ^&^ *P* < 0.05 compared with OGD/R + luteolin + BMS986299 group.

To study the role of NLRP3 in luteolin-mediated regulation of cell growth induced by OGD/R, NLRP3 was knocked down. When compared to the OGD/R group, the viability of SW10 cells was significantly increased after either knocking down NLRP3 or adding luteolin (*P* < 0.05). BMS-986299 significantly reduced the viability of OGD/R SW10 cells treated with luteolin, and the inhibitory effect was reversed by NLRP3 knockdown (*P* < 0.05, Figure 4a). Additionally, after knocking down NLRP3 or adding luteolin, the apoptosis proportion of SW10 cells in OGD/R declined significantly compared with the OGD/R group (*P* < 0.05). However, the addition of BMS-986299 to OGD/R SW10 cells treated with luteolin significantly increased the apoptosis rate of cells compared with OGD/R + luteolin group, and this increase was reversed by NLRP3 knockdown (*P* < 0.05, Figure 4b).

**Figure 5:**
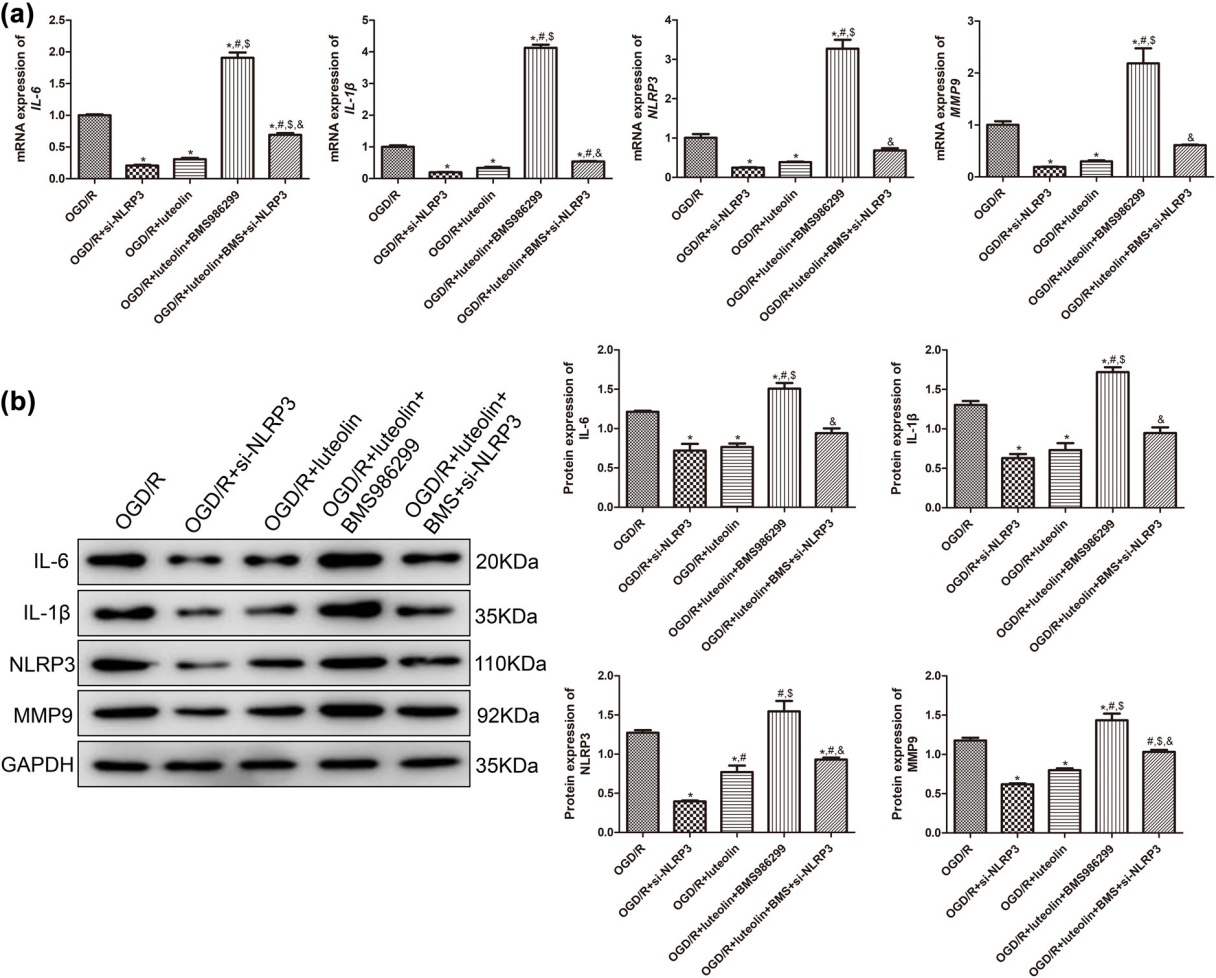
The roles of NLRP3 in luteolin regulating the expression of inflammatory factors induced by OGD/R. The expression of IL-6, IL-1β, NLRP3, and MMP9 after OGD/R, NLRP3 knocking down or treated with BMS-986299 detected by RT-qPCR (a) and Western blot (b). *N* = 3. *P < 0.05 compared with OGD/R group. ^#^ *P* < 0.05 compared with OGD/R + si-NLRP3 group. ^$^ *P* < 0.05 compared with OGD/R + luteolin group. ^&^ *P* < 0.05 compared with OGD/R + luteolin + BMS986299 group.

We also detected the expression of IL-6, IL-1β, NLRP3, and MMP9 in SW10 cells of OGD/R after knocking down NLRP3 or adding luteolin. The mRNA levels of these genes in OGD/R + si-NLRP3/luteolin groups were significantly lower than those in the OGD/R group (*P* < 0.05). However, when BMS-986299 was added into the OGD/R + luteolin group, the mRNA levels of these genes were elevated significantly, which could be reversed by NLRP3 knockdown (*P* < 0.05, Figure 5a). The protein expression trends of IL-6, IL-1β, NLRP3, and MMP9 in different groups were consistent with the mRNA expression trends (Figure 5b).

## 5 Discussion

Cerebral ischemia, characterized by OGD, initiates a cascade of cellular events culminating in neuronal injury and death. Currently, no approved treatment exists to mitigate neurological dysfunction [21]. Moreover, the availability of effective drugs for treating cerebral ischemia remains limited. Our study is the first to investigate the neuroprotective role of luteolin in cerebral ischemia. The findings indicated that OGD/R significantly decreased cell viability, increased apoptosis, and elevated the mRNA levels of *IL-6*, *IL-1β*, *NLRP3*, and *MMP9* in SW10 cells. Luteolin exerted neuroprotective effects by enhancing the cell viability-reducing apoptosis and inhibiting the expression of a series of inflammatory factors in injured SW10 cells following OGD/R. These protective effects were mediated through the regulation of the NLRP3/IL-1β signaling pathway and could be reversed by BMS-986299.

OGD/R is a widely used experimental model to mimic cerebral I/R injury, capable of inducing apoptosis and reducing the cell viability of neuronal cells [1,22]. Neuronal apoptosis frequently occurs in neurodegenerative diseases, leading to long-term alterations in brain function [22]. Our results demonstrated that luteolin could enhance OGD/R-induced cell viability and reduce apoptosis in SW10 cells, suggesting its neuroprotective properties of luteolin. A previous study has shown that the Naotaifang formula could relieve OGD/R-induced inflammation and ferroptosis through BMP6/SMADs signaling to regulate microglial M1/M2 polarization [23]. Edaravone is a well-known free radical scavenger with demonstrated neuroprotective effects in conditions like ischemic stroke. Yin et al. [24] demonstrated that edaravone could inhibit autophagy in neurons caused by OGD/R Another study manifested that edaravone-dexborneol, composed of edaravone and (+)-borneol, could significantly attenuate cerebral I/R injury both *in vitro* and *in vivo* via targeting OAT3/P-gp transporters for drug delivery into the brain [25]. Our *in vivo* experiments have clarified that luteolin could alleviate cerebral infarction, apoptosis, and pyroptosis in cerebral I/R injury. Therefore, the current *in vitro* experiments further confirmed that luteolin could improve OGD/R-induced injury by regulating cell viability and apoptosis.

Cerebral ischemia exacerbates brain injury by precipitating a robust inflammatory response [21]. An increasing body of evidence indicates that proinflammatory cytokines, such as IL-1β and IL-6, are the primary initiators of cerebral ischemic injury. Inflammation, moreover, plays a key role in the pathological progression of cerebral ischemic injury [26]. MMP9 has also been reported to regulate inflammation in various tissues and diseases [27,28]. MMP9 can activate inflammatory cells and facilitate the release of inflammatory factors, thereby further intensifying the inflammatory damage to brain tissue [29]. ROS is a crucial contributor of neuronal injury during OGD/R. The level of ROS was elevated by OGD/R but reduced by luteolin. Excessive production of ROS disrupts the redox equilibrium in cells, leading to lipid peroxidation, protein oxidation, and DNA damage, which will lead to cell dysfunction and death [30]. In addition, ROS can not only directly aggregate mitochondrial membrane and mitochondrial DNA but also increase blood-brain barrier permeability, activate NF-κB, and promote inflammation [30,31]. Our study indeed demonstrated that OGD/R induction triggered inflammation, increased the ROS levels, and up-regulated the expression of IL-6, IL-1β, NLRP3, and MMP9 in SW10 cells. However, luteolin suppressed their expression compared with the OGD/R group, suggesting that luteolin could reduce the release of inflammatory cytokines from injured SW10 cells caused by OGD/R.

It has been reported that the neuroprotective effects of luteolin are closely associated with ROS inhibition, mitochondrial function stabilization, and downstream transcription factors (e.g., NF-κB) [32,33,34]. A previous research showed that luteolin could protect cardiomyocytes from I/R-induced ferroptosis by inhibiting the accumulation of ROS and MDA [33]. Mitochondrial dysfunction is a hallmark of OGD/R injury, and luteolin could induce cell apoptosis through endoplasmic reticulum stress and mitochondrial dysfunction in neuroblastoma cells [35]. As a central regulator of inflammation, NF-κB activation exacerbates neuronal injury during OGD/R. The ability of luteolin to inhibit NF-κB signaling and reduce the expression of pro-inflammatory cytokines could further attenuate neuroinflammation and damage [36]. Mitochondrial dysfunction results in the accumulation of ROS and oxidized mtDNA within microglia, which leads to the activation and elongation of NLRP3 [37]. Additionally, NLRP3 is the best-studied inflammasome [38], playing a key role in the inflammatory response of I/R injury [39]. Interestingly, delayed NLRP3 expression has been detected in neurons during I/R injury [40]. NLRP3 has been reported to be the major contributor among the inflammasomes after transient middle cerebral artery occlusion (MCAO) in mice [41]. Therefore, NLRP3 inflammasome can act as a treatment target for cerebral I/R injury [42]. Moreover, NLRP3 inflammasome activation can up-regulate the IL‐1β expression and further promote the cascade of inflammation in the central nervous system, leading to the aggravation of nerve injury in patients with ischemic stroke [43,44,45]. Therefore, identifying interventions that can modulate the NLRP3/IL-1β signaling pathway holds great significance for neuroprotection and disease treatment. In this study, BMS-986299, an NLRP3 agonist, was found to significantly reduce the cell viability and increase the inflammatory factors expression of OGD/R SW10 cells treated with luteolin. This inhibitory effect was reversed by NLRP3 knockdown, indicating that the activity mediated by luteolin depends on the regulation of the NLRP3/IL-1β signaling pathway.

However, there are some limitations in this study. While the NLRP3/IL-1β signaling pathway is important, it could be beneficial to explore the additional pathways (such as NF-κB and mitochondrial function stabilization) that may contribute to luteolin’s effects. Second, further experiments, such as a CRISPR/Cas9-mediated knockout of NLRP3 in SW10 cells, as well as *in vivo* systems (interactions within the neural microenvironment or an *in vivo* MCAO model), should be conducted to investigate the specificity of luteolin’s actions on NLRP3. Additionally, the comparison analyses between luteolin and other neuroprotective agents (e.g., edaravone) need to be unearthed in the future.

In summary, we have demonstrated that luteolin alleviates the expression of inflammatory factors and protects neurons injured by OGD/R, which was mediated by suppressing the NLRP3/IL-1β signaling pathway. This study provides additional theoretical and data support for the potential clinical application of luteolin in mitigating neuron injury in cerebral ischemia.
